# Enzymatic debridement shall not modify the global strategy for mass burn events

**DOI:** 10.1186/s40779-022-00427-7

**Published:** 2022-11-26

**Authors:** Nicolas Donat, Thomas Leclerc, Stian Kreken Almeland

**Affiliations:** 1 Burn Treatment Centre, Percy Military Teaching Hospital, 92140 Clamart, France; 2 Val-de-Grâce Military Medical Academy, 75005 Paris, France; 3 Norwegian National Burn Center, Department of Plastic, Hand and Reconstructive Surgery, Haukeland University Hospital, 5021 Bergen, Norway

**Keywords:** Enzymatic debridement (ED), Burn care, Mass causality, Burn surgery

We read with interest the letter by Surowiecka et al. [1] about early burn wound excision in mass casualty events. We couldn’t agree more with their statement about the benefit of early burn wound excision. Still, we doubt whether applying this strategy to every patient during a mass burn event could be realistic. Of note, while there is an undisputed consensus that early burn wound excision is the gold standard of burn care, what ‘early’ actually means is still debated. Depending on the authors, the corresponding time limit typically varies from 24 h to a few days [2, 3].

Regarding the enzymatic debridement (ED) role in massive burn casualties, we are not convinced of its particular benefit. Indeed, it could spare surgical and operating room time for excision procedures. However, its correct use is known to have a learning curve upon implementation and is limited to specialized burn teams [4]. As such, burn centers and teams able to provide this highly trained resource will likely be saturated in a massive burn casualty event. ED would not automatically relieve Burn Intensive Care Unit saturation and does not necessarily ease their workload as they would have to spend time and resources on applying bromelain enzyme and the resulting added number of dressing changes in the ward. Although the operating room might be physically relieved from the workload, the burn ward’s anaesthetist, nursing, and surgical staff would be very much burdened by an intense workload [4].

We agree with the idea that ED would probably be useful for mild to intermediate severity burns that do not require Intensive Care Unit or intermediate level care and can be managed in a surgical ward. Even for those patients, ED should not be considered a magic bullet as the workload may only be transferred to another activity. Indeed, wound care with ED can be complex and painful and may require general anaesthesia or sedation. Such advanced care cannot easily be provided in a random surgical room.

Severe burns require very extensive hospital stays, typically more than one hospital day per percent total burn surface area, and are normally cared for in highly specialized burn centers [5]. There is a limited number of such specialized burn centers in each country. The key challenge in burn mass casualty incidents is the saturation of both specialized facilities and non-specialized facilities where casualties have been initially admitted. For the former, ED may be a valuable tool to help optimize operating room utilization, yet with the aforementioned limits. For the latter, ED is unlikely to be of help and could even worsen the resulting local chaos. Introducing unfamiliar care protocols in a disaster setting is not recommendable. On the contrary, it should rather be a priority to keep the chosen care methods in a disaster as close to normal-day routine care as possible. Sound disaster management principles applied to such situations advocate early patient transfer to burn centers. This should be the optimal way of achieving early and adequate burn wound excision, surgical or enzymatic, without impairing further patient management and increasing the burden on first-line non-specialized hospitals.

From a military point of view, we do not know about any health service policy that implements early excision for combat wounds, i.e., burn excision by forward surgical teams. Seemingly, they have all implemented an early evacuation (within 3 d) to homeland facilities where excision will be performed [6]. ED couldn’t be used in this particular setting until patients are admitted to homeland facilities, limiting the potential benefit. Once again, the bromelain enzyme could hardly be applied for military casualties for several reasons. No specially trained teams are available in field hospital theatres, nor could ED be applied by dedicated burn evacuation teams during the flight without considerable safety concerns.

We want to stress that the overall strategy for mass burn casualty events is to evaluate patients and evacuate the most severe patients whose medical condition is compatible with medical evacuation as soon as possible, best in the first 72 h. This strategy allows to spare the most scarce national resources: the specialized burn centers with highly trained surgical and intensivist teams [7, 8].

The potential benefit of ED seems to be limited to a small group of casualties and should, therefore, not modify the global strategy for mass burn events.

## Data Availability

Not applicable.

## Abbreviation

ED: Enzymatic debridement
